# What drivers phenotypic divergence in *Leymus chinensis* (Poaceae) on large-scale gradient, climate or genetic differentiation?

**DOI:** 10.1038/srep26288

**Published:** 2016-05-19

**Authors:** Shan Yuan, Linna Ma, Chengyuan Guo, Renzhong Wang

**Affiliations:** 1 State Key Laboratory of Vegetation and Environmental Change, Institute of Botany, the Chinese Academy of Sciences, 20 Nanxincun, Xiangshan, Beijing, 100093, China

## Abstract

Elucidating the driving factors among-population divergence is an important task in evolutionary biology, however the relative contribution from natural selection and neutral genetic differentiation has been less debated. A manipulation experiment was conducted to examine whether the phenotypic divergence of *Leymus chinensis* depended on climate variations or genetic differentiations at 18 wild sites along a longitudinal gradient from 114 to 124°E in northeast China and at common garden condition of transplantation. Demographical, morphological and physiological phenotypes of 18 *L. chinensis* populations exhibited significant divergence along the gradient, but these divergent variations narrowed significantly at the transplantation. Moreover, most of the phenotypes were significantly correlated with mean annual precipitation and temperature in wild sites, suggesting that climatic variables played vital roles in phenotypic divergence of the species. Relative greater heterozygosity (H_E_), genotype evenness (E) and Shannon-Wiener diversity (I) in western group of populations suggested that genetic differentiation also drove phenotypic divergence of the species. However, neutral genetic differentiation (F_ST_ = 0.041) was greatly lower than quantitative differentiation (Q_ST_ = 0.199), indicating that divergent selection/climate variable was the main factor in determining the phenotypic divergence of the species along the large-scale gradient.

It has long been recognized that geographic variation in phenotypes of an organism can include both environmental and genetic components, thus determining the mechanisms that cause such variation is one of the basic objectives of ecological and evolutionary studies^1^. Plant population responding to environmental changes depends on the competition of divergent natural selections causing local adaptation versus gene flows leading to alleviation of quantitative differentiation^2^,^3^. Divergent selections contribute to local adaptation and intraspecific differentiation^4^,^5^,^6^. Moreover, the molecular genetic differentiation also attribute to the phenotypic divergence at a species-wide scale^7^. In contrast, gene flows are generally considered to maintain genetic homogeneity within a species by seed and pollen distributions^8^,^9^. However, some studies have proposed that moderate levels of migration could help purge deleterious mutations and replenish genetic variability, enhancing natural selection and counteracting drift^10^,^11^. Despite the fundamental importance of studying natural genetic variation and the describing patterns of phenotypic divergence among different environments^12^,^13^, few studies have jointly investigated the relative contributions of environmental and molecular genetic factors on quantitative differentiation across a species range. This knowledge is essential for formulating generalization regarding mechanisms for plant adaptation and evolution with global changes.

To determining the relative functions of environmental and genetic factors, the comparison of quantitative differentiation in common garden experiments (Q_ST_) and neutral genetic differentiation (F_ST_) among populations could be effective^11^,^12^,^14^. The quantitative differentiation in wild samplings (P_ST_) was the analogue of Q_ST_, and also informative to compare the relative influence of genetic adaptation, phenotypic plasticity and genetic drift as causes of population differentiation^14^. Three outcomes are possible in natural populations: If P_ST_/Q_ST_ > F_ST_, phenotypic divergence exceeds neutral expectation and is likely to have been caused by directional selection for environmental adaptability. For instance, the aridity gradient was demonstrated to be the main driving factor for the population differentiation in molecular markers was much lower than in quantitative traits in wild barley^11^. In cases P_ST_/Q_ST_ ≈ F_ST_, the inference is that phenotypic divergence could have been achieved by molecular differentiation among subpopulations. If P_ST_/Q_ST_ < F_ST_, this pattern is suggestive of uniform selection across the populations^14^. Divergent selection for environmental variability usually drives significant quantitative differentiation along the broad scale gradient^12^,^14^.

*Leymus chinensis* (Trin.) Tzvel., a perennial rhizomatous C_3_ cosmopolitan grass, is widely distributed at eastern end of Eurasian steppe zone, from western part of the Northeast Plain to eastern part of the Mongolian Plateau, China. The xerophytic traits, *e.g*. thick rhizome systems and high plasticity in leaf traits, enable the species to successfully tolerate and/or resist drought when soil moisture is less than 4% in dry seasons in desert steppes^15^. Widely distributions of the cosmopolitan grass make it an ideal plant species for studying adaptation under large-scale environmental changes. Responses of the species to climatic variations have been well documented on population density, anatomical, morphological and physiological plasticity^16^,^17^,^18^. However, the driving mechanism of the phenotypic divergence in the species along large-scale gradient has not yet been addressed, this is essential for understanding the evolutionary trajectory of plant species under global climate change. We hypothesize that both environmental changes and molecular genetic differentiation drove the phenotypic divergence within the species. The objective of the present study was to compare the functions of environmental adaptation and neutral genetic differentiation in phenotypic divergence of *L. chinensis*, aiming to better understand the driving mechanism of plant phenotypic variations and adaptive strategies to stress conditions under broad scale climate change. Furthermore, our specific aims were to examine: (1) the obvious divergence of quantitative traits in *L. chinensis* and their relationships with climate variables along the gradient; (2) the roles of neutral genetic differentiation on phenotypic divergence.

## Results

### Demography and morphological phenotypes

Population demography and morphological phenotypes in the species varied significantly along the gradient. Total shoot densities of *L. chinensis* at the east end (01 site, 903.4 m^−2^) were more than 3-fold of that at the west end (18 site, 283.4 m^−2^) (*P* < 0.01, Table 1; Supplementary Fig. S1; Supplementary Table S1) and significantly and positively correlated with mean annual precipitation (MAP) (*P* < 0.05), mean annual temperature (MAT) (*P* < 0.05) and soil water content (SWC) (*P* < 0.01), but negatively correlated with elevation (*P* < 0.01). Vegetative shoot densities and reproductive shoot densities from eastern sites (01–10 sites, 567.2 m^−2^ and 123.5 m^−2^) were nearly 2-fold as those from western ones (11–18 sites, 306.6 m^−2^ and 63.6 m^−2^, *P* < 0.01, respectively). The vegetative shoot densities were significantly and positively correlated with MAP (R^2^ = 0.42, *P* < 0.05) and SWC (R^2^ = 0.59, *P* < 0.01). The reproductive shoot differentiation ratio (RSDR) in desert steppes (17–18 sites, 0.077) was 57.9% lower than that in meadows (01–10 sites) and typical deserts (11–16 sites, 0.183) (Supplementary Table S1).

**Figure 1:**
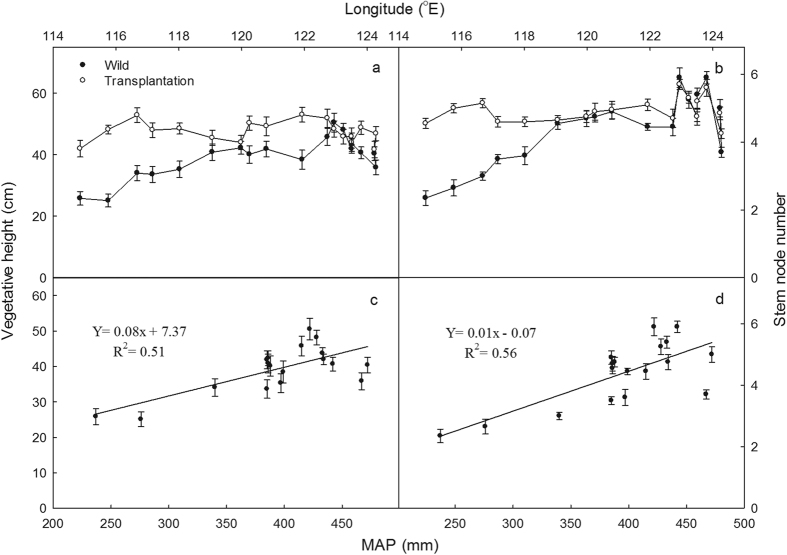
Vegetative shoot height (**a**), stem node numbers (**b**) in wild sites and transplantation and their regressions with mean annual precipitation (MAP) (**c**,**d**) in *L. chinensis* along the large-scale gradient.

In wild sites, vegetative shoot heights dropped sharply from 50.5 cm (07 site) to 25.1 cm at the west end (Fig. 1a). Correspondingly, stem node numbers diminished from average 4.9 in meadows to 2.5 in desert steppes (Fig. 1b). Vegetative shoot heights were significantly correlated with stem node numbers (R^2^ = 0.787, *P* < 0.01). Both vegetative shoot heights and stem node numbers were significantly and positively correlated with MAP in wild sites (*P* < 0.05) (Fig. 1c,d), MAT (*P* < 0.05), but negatively with elevation (*P* < 0.01). Average reproductive shoot height was 40.1 cm in desert steppes, reduced by 34.7% comparing to 61.4 cm in meadows (Supplementary Table S2). In transplantation, however, the corresponding 18 populations did not exhibit significant variation in vegetative shoot heights, and that was on average (47.2 cm) 18.3% higher than those from wild sites (39.9 cm, *P* < 0.01) (Fig. 1).

Leaf lengths at meadows (20.7 cm) were 12.6% higher than that at typical steppes and desert steppes (18.6 cm) in wild sites (*P* < 0.05) (Supplementary Table S2). On the contrary, the leaf lengths of corresponding populations from eastern sites (26.4 cm) were not significantly different from the western ones (28.6 cm) at transplantation (*P* > 0.05). Average leaf length at transplantation (27.4 cm) was 31.1% higher than that of wild sites (20.9 cm, *P* < 0.05) (Supplementary Table S2). Leaf lengths in wild sites were significantly and positively correlated with MAP and MAT (*P* < 0.05) (Supplementary Fig. S2a,b), but negatively correlated with elevation (*P* < 0.01) (Supplementary Fig. S2c).

Unlike leaf length, leaf width in western sites (0.68 cm) was 18.8% higher than eastern ones (0.57 cm) in wild samplings (*P* < 0.01), but there was no significant difference between corresponding eastern (0.65 cm) and western (0.68 cm) populations at transplantation (*P* > 0.05) (Supplementary Table S2). Leaf area significantly dropped from east to west, with the maximum 9.4 cm^2^ (07 site) and minimum 5.1 cm^2^ (14 site) in wild sites. Average leaf area at transplantation (11.4 cm^2^) was 59.5% higher than that from wild sites (7.2 cm^2^, *P* < 0.01) (Supplementary Table S2). Leaf mass per area (LMA) in wild samplings (11.09 mg cm^−^) was 22.4% higher than that of transplantation populations (9.06 mg cm^−2^, *P* < 0.01). LMA was significantly and negatively correlated with MAP and SWC, but positively with elevation in wild sites (*P* < 0.05).

Average seed weight and spike length from meadow and typical steppe sites were 54.3% (*P* < 0.01) and 51.3% (*P* < 0.01) greater than that from the desert sites, respectively. Seed weights and spike lengths were significantly and positively correlated with MAP and MAT in wild sites (*P* < 0.01) (Supplementary Fig. S2d–f; Supplementary Table S2).

### Physiological adjustments

**Figure 2:**
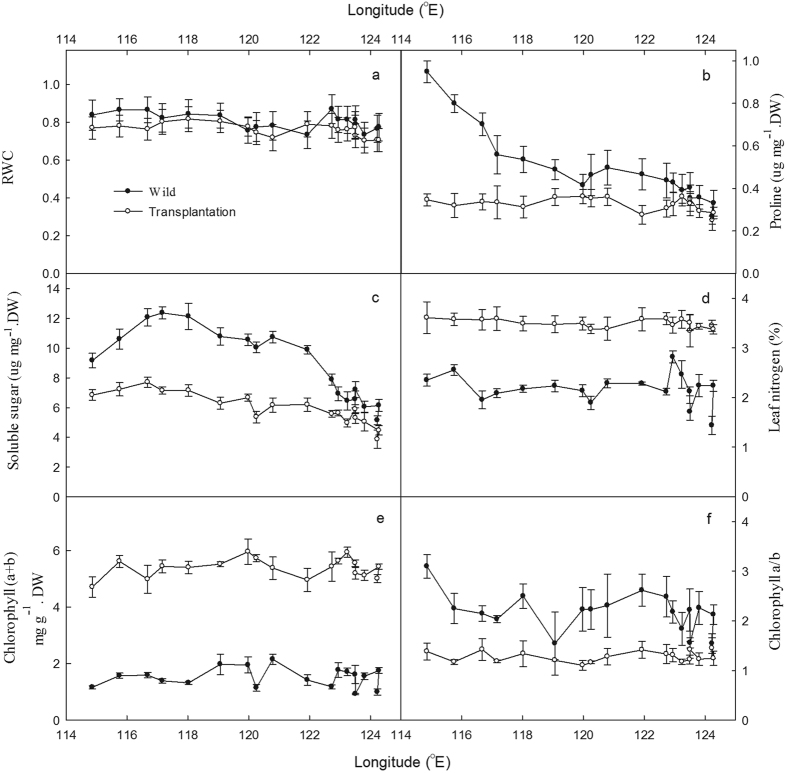
Physiological phenotypes of relative water content (RWC) (**a**), proline (**b**), soluble sugar (**c**), leaf nitrogen (**d**), chlorophyll (a + b) (**e**), and chlorophyll a/b (**f**) in wild sites and transplantation.

Physiological adjustments/phenotypes of the species differed significantly along the broad gradient (Fig. 2). Relative water content (RWC) maintained a relatively high level (average 80.5%) in wild sites, dropped by 6.4% at transplantation (average 75.3%, *P* < 0.05) (Fig. 2a). RWC was significantly and positively correlated with MAP and MAT in wild sites (*P* < 0.05).

Both proline and soluble sugar contents of the species increased from the east to the west along the gradient. Proline contents from desert steppe sites (0.874 ug mg^−1^.DW) were about 2-fold of that in meadows and typical steppes (0.444 ug mg^−1^.DW, *P* < 0.01) (Fig. 2b). However, soluble sugar contents showed different patterns, at first increased, maximizing at 117°E (site 15, 12.363 ug mg^−1^.DW), then decreasing at the west end, and the average value in typical steppes (11.320 ug mg^−1^.DW) were 46.4% higher than those in meadows and desert steppes (7.731 ug mg^−1^.DW, *P* < 0.01), respectively (Fig. 2c). At transplantation, there was no significant difference in proline contents between the western and eastern populations (*P* > 0.05), whereas soluble sugar content of western sites was 18.1% higher than that of eastern ones (*P* < 0.05). Proline and soluble sugar contents reduced by 33.7% and 35.7% in transplantation compared with their wild populations (*P* < 0.01) (Fig. 2). Proline and soluble sugar contents were significantly and negatively correlated with MAP (*P* < 0.01) (Fig. 2b,c) and SWC (*P* < 0.01) along the gradient.

Leaf nitrogen (N) contents and Chlorophyll (Chl) (a + b) exhibited slight variation along the gradient (Fig. 2d,e). Leaf N contents at transplantation (3.49%) was 60.8% higher than that of wild populations (2.17%) (*P* < 0.01). Average Chl (a + b) content in transplantation (5.388 mg g^−1^) was more than 3-fold compared with the wild samples (1.511 mg g^−1^, *P* < 0.01) (Fig. 2e). Unlike Chl (a + b) contents, Chl a/b ratio increased from 1.543 at site 01 to 3.097 at site 18 (Fig. 2f) and significantly and negatively correlated with MAP (*P* < 0.05) along the gradient. Average Chl a/b ratio (1.281) in transplantation dropped by 41.1%, compared with the wild populations (2.176, *P* < 0.01) (Fig. 2f).

### Molecular analysis

Genetic diversities in eastern sites (expected heterozygosity (H_E_): 0.628, genotype evenness (E): 0.098 and Shannon-Wiener diversity (I): 1.105) were significantly higher than that in western ones (11–18 sites, H_E_: 0.579, E: 0.085 and I: 0.972, *P* < 0.01), with average of 18 population H_E_ = 0.606, E = 0.092, I = 1.046 (Supplementary Table S3). Analysis of molecular variance (AMOVA) revealed significant genetic differentiation among inferred populations, accounting for 7.28% of the total molecular variation (*P* < 0.01). There were 6.16% differentiation between eastern - western sites (*P* < 0.01) and 4.44% among three vegetation types (meadow, typical steppe and desert steppe, *P* < 0.01), but not significant differentiation between soil pH groups (nearly neutral and alkaline, *P* > 0.05, Table 2).

**Figure 3:**
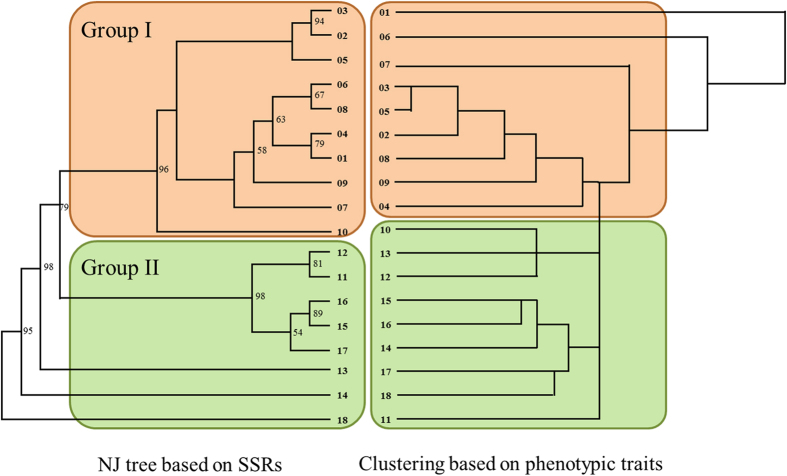


NJ clustering divided the 18 populations into two genetically differentiated groups with high bootstrap supports (Fig. 3), including group I: all of the meadow sites (01–10), group II: mixed with typical steppe and desert steppe sites (11–18). Clusters based on 16 phenotypes (vegetative and reproductive shoot heights, leaf length, leaf width, leaf fresh weight, leaf turgid weight, leaf dry weight, leaf area, node numbers, spike length, seed weight, proline, soluble sugar, leaf N content, Chl (a + b) and Chl a/b) were slightly different from NJ tree, with group I covering 9 sites (01–09 sites) and group II containing the other 9 sites (10–18 sites) (Fig. 3). STRUCTURE divided genetic variation into two main geographic groups (K = 2) and this consistent with NJ tree: (1) eastern group (site 01–10) and (2) western group (site 11–18). (Supplementary Fig. S3; Supplementary Fig. S4). Pairwise genetic distance^19^ ranged from 0.003 (16 and 17 site populations) to 0.382 (02 and 18 site populations) with average 0.166, was significantly correlated with geographic distance (km) (*P* < 0.01) (Supplementary Table S4; Supplementary Fig. S5). Low F_ST_ values were detected among pairs of populations: pairwise F_ST_ varied from 0.009 (16 and 17 site populations) to 0.100 (02 and 11 site populations), with average 0.041 (Supplementary Table S5). Mean pair-wise population gene flow (pairwise Nm) was 5.791 based on average F_ST_ (Supplementary Table S5).

### Q_ST_–F_ST_ comparison

**Figure 4:**
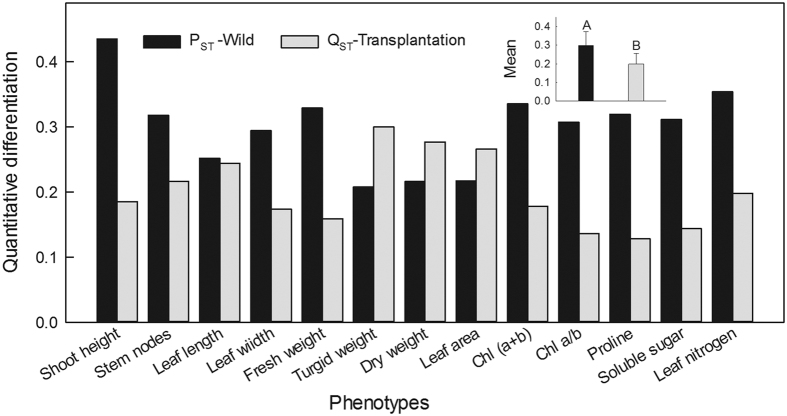


There was a more pronounced level of differentiation in phenotypes (mean Q_ST_ = 0.199 at transplantation) for the 13 statistic traits (vegetative shoot height, leaf length, leaf width, leaf fresh weight, leaf turgid weight, leaf dry weight, leaf area, node numbers, proline, soluble sugar, leaf N content, Chl (a + b) and Chl a/b) than that of neutral markers (F_ST_ = 0.041 for 15 microsatellite loci) among the 18 populations (Fig. 4). Average quantitative differentiation in wild populations (P_ST_ = 0.299) was 49.5% higher than Q_ST_ value (0.199) in transplanted ones (*P* < 0.01).

## Discussion

Widely distributed species often exhibit considerable variations in morphological, physiological and other fitness-related characters in complex environments where climate conditions fluctuated^2^,^20^. For plants, ecologically important traits display clinal patterns of population divergence under environmental variations^6^,^21^. These patterns can provide strong evidence for spatially varying selection across environmental gradients or result from neutral genetic processes^9^. Therefore, evaluating the relative importance of environmental and genetic functions in determining the quantitative differentiation among populations is essential for our ability to predict how plants will respond and adapt to global climate change^22^. Along the large-scale gradient in northeast China, drought is one of the major environmental factors affecting plant survival, distribution, resource partitioning, growth and reproduction^17^,^18^. The significant decrease in shoot densities, shoot height, leaf area, seed weight and spike length, and increase in leaf width and LMA (Fig. 1; Supplementary Table S1; Supplementary Table S2) among *L. chinensis* populations from east to west exhibited more obvious xerophil-liked phenotypes, suggesting climate variables are the critical driver factors for phenotypic divergence of the species along the large-scale gradient. Relative less shoot densities, shoot heights and leaf area in dry conditions may reduce water loss by plant transpiration^16^,^17^, while higher LMA was proved to be a main adaptive strategy to droughts for higher LMA, which was positively related with photosynthetic tissue per area and investment in structural tissues, contributing to higher tolerance in unfavorable conditions^15^,^17^. The significant correlations of phenotypes (*e.g*. shoot densities, shoot heights and LMA) with MAP indicated that obvious divergence of quantitative traits in *L. chinensis* was strongly related with moisture conditions along the longitudinal gradient (Fig. 1; Supplementary Fig. S2). This was also supported by the less variation in morphological phenotypes of 18 populations with sufficient water supply and non-saline-alkali soil at transplantation (Fig. 2; Supplementary Table S2). Moreover, relatively less correlation between phenotypes and soil properties (soil pH and soil N contents) eliminated the soil alkaline and soil nutrient as the main driven factor in *L. chinensis* divergence at large-scale gradient (Figs 1 and 2). Other studies on *Triticum dicoccoides*^23^, *Boechera holboellii*^24^, *Solanum lycopersicum*^25^ have demonstrated that ecologically important phenotypes were significantly tied to the climate variations as well.

Physiological regulations in *L. chinensis* manifested its higher tolerance to drought stress. Increasing proline, soluble sugar contents and RWC of the species from east to west suggested its capacity for dehydration tolerance and the osmotic adjustments were enhanced with the aridity along the broad scale gradient (Fig. 2). Free proline accumulation in plant leaves is one of the most common and direct biochemical responses to water deficit, and enabled cell to maintain turgor and resulted in relative higher RWC for plants during drought and saline stresses^26^,^27^. Relative higher RWC of the species in typical steppes and desert steppes demonstrated that proline had taken part in osmotic adjustment (Fig. 2a). More soluble sugar contents in meadows and typical steppes indicated the effective accumulation under moderate drought, while lower contents in desert steppes were likely affected by severe water deficit (Fig. 2c). Through Chl (a + b) contents maintained high level along the gradient, the dramatically reduced leaf area could decrease the photosynthetic materials, resulting in the less photosynthetic productions, *e.g*. soluble sugar (Fig. 2e; Supplementary Table S2). The significantly negative correlations between osmotic adjustment substance (proline and soluble sugar) contents and MAP and SWC (Fig. 2b,c), as well as the positive correlation between Chl a/b and MAP in wild sites (Fig. 2f), showed that water deficit possibly drove the physiological differentiation along the gradient. Relative less variation in drought-responsive physiological adjustments (proline and soluble sugar contents) at transplantation also supported that the precipitation was one of the major driving factors in these physiological phenotypes of *L. chinensis* (Fig. 2). Concostrina-Zubiri *et al*.^28^ found a general increase of functional diversity with increasing precipitation of the lichen-dominated biological soil crusts in semiarid steppes. These findings (*e.g*. the decrease of shoot densities, shoot height, leaf size, seed weight, the increase of RWC and proline from east to west, as well as the significant correlations between these phenotypes and climate variables) supported our first hypothesis that the phenotypic divergence of *L. chinensis* was strongly driven by climate forces along the large-scale gradient.

The significant molecular differentiation was detected in western-eastern sites by NJ clustering and Bayesian assignment results among *L. chinensis* populations (Fig. 3; Supplementary Fig. S4). Relative greater genetic differentiation in western group of populations suggested that genetic differentiation of *L. chinensis* was not only related to the large-scale geographic distribution, but also to environmental heterogeneity, especially the strong droughts in the western sites (Table 2). Moreover, the eastern-western division of genetic diversities (H_E_, E and I values) and genetic differentiations were probably attributed to the uplift of Mongolian plateau at site 11: elevation suddenly lifted from 276 m to 421 m, SWC dropped from 12% to 9% and vegetation types transformed from meadows (site 10) to typical steppes (site 11) (Table 1). Both environmental and geographical selections could contribute to the discrete genetic differentiation among natural populations of *L. chinensis* along the broad gradient^17^,^29^.

Selection and drift can promote genetic differences if populations are sufficiently isolated, however, gene flow prevents differentiation and local adaptation^30^,^31^. Average pairwise F_ST_ (0.041) from SSR datasets (Supplementary Table S5) in *L. chinensis* was significantly lower than that observation from random amplified polymorphic DNA (RAPD, F_ST_ = 0.146)^32^ and also much lower than that of other plants (*e.g*. average F_ST_ = 0.25 for 13 widespread species; F_ST_ = 0.22 for 23 outcrossing species^33^. On the contrary, the sufficient gene flows among 18 populations (average Nm = 5.791, calculated by pairwise F_ST_ values, Supplementary Table S5) suggested migrants per generation were possibly facilitated by wind pollination with light seeds and outcrossing system for self-incompatibility in this species^34^,^35^. Generally, gene exchange was considered to homogenize populations and counteract divergence, however, gene flow from adjacent populations (*e*.*g*. in similar environment) via seeds had a positive effect on the population fitness by bringing in already selected genotypes, thus exaggerated the genetic differentiation^2^. Some previous studies have proposed that moderate levels of migrations may provide new genetic materials for local selection to act upon and enhance natural selection^36^,^37^. To some degree, the roles of gene flow are not completely opponents to divergent natural selection in traditional view, but more like an “assistant” character for *L. chinensis* population differentiation between adjacent populations under similar environments.

Q_ST_ (0.199) estimated from *L. chinensis* quantitative traits at transplantation were more than 4-fold of the F_ST_ values (0.041) from microsatellite loci (Fig. 4), suggesting that role of divergent climatic selection greatly exceeded the neutral genetic differentiation on these phenotypes. The *L. chinensis* populations are locally adapted in their wild habitats and their adaptability was strongly enhanced by the obvious xerophil-liked traits and biochemical strategies, which were stimulated by the precipitation and temperature reductions from east to west along the gradient. Moreover, the Q_ST_ in transplantation populations was about 1/3 less than the P_ST_ (0.299) in wild populations, demonstrating that quantitative differentiation in *L. chinensis* was significantly affected by the environmental changes (Fig. 4). There were both theoretical and empirical researches focusing on climatic gradients in generating differentiation and even speciation through divergent selections^38^,^39^,^40^. For instance, the results of Q_ST_–F_ST_ comparison on wild barley showed that population differentiation in molecular markers was much lower than in quantitative traits, in agreement with adaptive selection along the aridity gradient^11^. Higher level of population differentiation for Q_ST_ (0.535) than F_ST_ (0.003) were observed in maize, suggesting quantitative traits appear to be under strong divergent selection^41^. Furthermore, neutral genetic divergence increased with environmental heterogeneity, which was positively correlated with geographic distance^2^. The results of Q_ST_–F_ST_ comparison and neutral molecular differentiation supported our second hypothesis that neutral genetic differentiation played an important role in phenotypic divergence, but divergent selection/climate variables was the main factor in determining the phenotypic divergence of the species along the large-scale gradient.

Our findings suggested that climate changes (precipitation and temperature) and molecular genetic differentiation both drove quantitative differentiations of *L. chinensis* across the longitudinal gradient. This research highlighted the combination of phenotypes divergence and genetic differentiations and systematical analysis of quantitative traits in both wild sites and transplantation, providing reliable information about the driving factors for the phenotypic divergence which could not been addressed in wild (to detect the phenotypic plasticity in broad scale field)^42^ or common garden experiment (to identify differences in quantitative traits between different genotypes)^43^ alone.

## Materials and Methods

### Study sites

The experiment was conducted in natural *L. chinensis* grasslands and greenhouse from 2012 to 2014. Eighteen populations/sites were selected along a longitudinal gradient in northern China, ranging from 43°24′ to 44°36′ N, 114°34′ to 124°16′ E, with approximate 900 km west-to-east (Table 1). The MAT ranges from 1.7 °C to 5.1 °C. Moisture gradient is very steep, with MAP varying from 237 mm in the west to 467 mm in the east (Table 1). Due to the steep increase of precipitation from the west to the east, vegetation types transform gradually from desert steppes and typical steppes in the west to meadows in the east^44^: meadows (sites 01–10), typical steppes (sites 11–16) and desert steppes (sites 17–18). These sites had not been grazed, ploughed, fertilized or burned for at least 5 years prior to 2012, although transient floods may have occurred in the eastern meadows. A more detail description of the sampling locations, climate and soil properties can be found in Table 1.

### Demographical and morphological phenotypes

At least two ha of typical native *L. chinensis* grassland was selected for plant sampling at each site. Samplings were carried out in eight randomly located 1 × 1 m^2^ quadrants at each site in 2012 and 2013, respectively. Within each quadrant, reproductive shoot and vegetative shoot densities were counted (m^−2^). RSDR refers to the reproductive shoot density as a percentage of the total population density^16^. Forty shoot heights (vegetative and reproductive, respectively) and stem node numbers were measured randomly in each site. Forty spikes per site were collected randomly to measure spike length and seed weight in wild populations.

Twenty 2^nd^ fully expanded leaves (from the shoot top, hereafter) in each population were sampled randomly from mature plants. Leaf traits (*e.g*. leaf length, width and area) were measured using a flatbed scanner connected to a personal computer running image analysis software. Leaf samples were placed in perforated paper bags and oven-dried at 80 °C for 24 h and then weighed to measure dry leaf mass. LMA was expressed as leaf dry mass per area (mg cm^−2^).

### Transplantation experiments

Underground rhizomes of each population were taken from 10 monoliths (25 × 25 × 30 cm^3^) from each site and transplanted into a greenhouse in Institute of Botany, the Chinese Academy of Sciences (Beijing). Rhizomes were placed into pots (25 cm diameter) with near-neutral organic soil (pH 7.3–7.5). The temperature and humidity in the greenhouse were controlled between 20–30 °C and 50–60%, respectively. Each pot was watered to maintain the soil water content above 20% with running water every other day in the first month, then 100 ml twice a week. After 3 months of growth, leaves were collected for morphological and physiological measurements.

### Physiological phenotypes

Twenty 2^nd^ fully expanded leaves in each site were sampled randomly from mature plants at about 9:00 am. Fresh weight (FW), turgid fresh weight (TW, overnight soaking) and dry weight (DW) were measured. Relative water content^45^


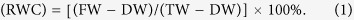


For proline, soluble sugar, leaf N and Chl content analyses, leaves from wild sites and transplantation were oven-dried under 105 °C for 20 min, then at 80 °C for 24 hr to a constant weight and grounded to pass through a 100-mesh screen. Proline content was estimated by the acid-ninhydrin method^46^. Soluble sugar content was measured using the anthrone-sulfuric acid method^47^. Leaf N content was determined by Kieldahl method^48^. Chl a and Chl b contents were extracted by 95% (v/v) ethanol and analyzed by conventional spectroscopic methods^49^.

### Microsatellite analysis

Thirty leaves from independent patches were collected randomly at each site then immediately dried in the silica gel and a total of 540 individuals were sampled. Genomic DNA was extracted and the concentration was examined by agarose gel electrophoresis. For microsatellite analysis, 80 pairs of primers were selected for candidates from previous publications^50^,^51^,^52^. Fifteen pairs of primers were identified to be suitable polymorphisms by the pre-experiment with 18 random individuals (Supplementary Table S6). The detail microsatellite protocol and analysis were according to the literature^53^.

### Soil properties and climate data

The soil samples were taken with a cylindrical soil sampler (5 cm inner diameter, 15 cm length) for the 0–15 cm layer. Among 7 and 14 soil samples were collected randomly per site (100 × 100 m) for determination of soil properties. Gravimetric soil water content was measured by oven-drying samples at 105 °C for 24 h, and the equation for calculating the


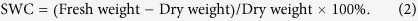


Soil pH was determined by a Model HS-3C pH meter (Shanghai Rex Instruments Factory, China). Soil N contents were measured using Kieldahl method^46^. Climate data was provided by the National Meteorological Information Center of China Meteorological Administration (http://www.worldclim.org/).

### Statistical analyses

One-way analysis of variance (ANOVA) was performed to analyze the differences in morphological and physiological phenotypes among the 18 populations. Linear regression analyses were performed to evaluate the relationship of quantitative traits with environmental factors (MAP, MAT, SWC, soil N, soil pH and elevation). All statistics for phenotypes were analyzed using SPSS 20.0 for windows^54^.

Population genetic analyses were performed on microsatellite dataset scored by the program GeneMarker v2.2.0^55^. Descriptive statistics including H_E_, I, and E and population pairwise genetic distances were calculated with Atetra program^56^. To assess the population genetic structure, following analyses were conducted: 1) Nei’s genetic distance was used to generate the Neighbor-joining tree with Neighbor of Phylip v3.63^57^. 2) The Mantel test was assessed on genetic distance and geographic distance (km) matrices by TFPGA 1.3^58^. 3) Population genetic structure was inferred by a Bayesian method using STRUCTURE 2.3.4^53^,^59^. 4) AMOVA was performed to quantify the genetic variance among populations with Arlequin 3.5^60^. 5) Pairwise F_ST_ values were evaluated by Polysat package running on the R platform^61^. Gene flow (the mean number of immigrants) was calculated by following:


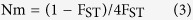


Quantitative differentiation was estimated by the formula described below^62^.


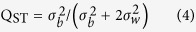


where 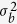 and 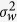 are between- and within- population components of variance. The partitioning of phenotypic variance within and between populations was appraised using ANOVA by SPSS 17.0. P_ST_ is a Q_ST_ analogous that estimated from the wild sampling data^12^,^14^.

## Additional Information

**How to cite this article**: Yuan, S. *et al*. What drivers phenotypic divergence in *Leymus chinensis* (Poaceae) on large-scale gradient, climate or genetic differentiation? *Sci. Rep.* **6**, 26288; doi: 10.1038/srep26288 (2016).

## Supplementary Material

Supplementary Information

## Figures and Tables

**Table 1:** Sample locations, vegetation types, climate and soil properties.

**Location**	**No.**	**Latitude**	**Longitude**	**Vegetation**	**ELE (m)**	**MAP (mm)**	**MAT (°C)**	**Soil pH**	**Soil N (%)**	**SWC (%)**
Wulantuga	01	44°28.722′	124°13.671′	Meadow	291	472	5.1	8.5	0.20	23
Chaganhua	02	44°35.475′	124°16.543′	Meadow	202	467	5.1	8.5	0.20	24
Wulanaodu	03	44°35.999′	123°48.219′	Meadow	152	442	5.3	8.1	0.16	22
Yaojingzieast	04	44°35.604′	123°30.259′	Meadow	165	433	5.4	8.4	0.23	23
Yaojingziwest	05	44°34.413′	123°29.887′	Meadow	159	434	5.4	7.8	0.16	17
Taipingchuan	06	44°21.323′	123°14.136′	Meadow	150	428	5.6	8.6	0.13	18
Jiamatu	07	44°01.654′	122°56.521′	Meadow	149	422	6.0	8.2	0.17	14
Baolongshan	08	43°58.237′	122°43.677′	Meadow	156	415	6.0	7.9	0.13	13
Molimiao	09	43°34.277′	121°55.444′	Meadow	179	399	6.3	8.4	0.12	12
Shaogen	10	43°38.831′	120°47.316′	Meadow	270	385	6.8	8.0	0.11	12
Tianshaneast	11	43°50.442′	120°15.015′	Typical steppe	413	388	5.8	8.2	0.19	9
Tianshanwest	12	43°50.277′	119°58.587′	Typical steppe	513	386	5.8	8.3	0.19	8
Xingfuzhilu	13	43°42.818′	119°03.749′	Typical steppe	735	386	4	8.4	0.11	8
Xinchengzi	14	43°14.175′	118°01.237′	Typical steppe	919	397	3.5	7.7	0.15	10
Dalainuori	15	43°16.157′	117°09.659′	Typical steppe	1309	385	1.3	8.1	0.14	8
Aqiwula	16	43°33.691′	116°40.619′	Typical steppe	1239	340	1.3	8.8	0.15	7
Dabuxiletu	17	43°55.880′	115°45.252′	Desert steppe	1158	276	1.4	7.8	0.10	5
Baogedawula	18	43°59.550′	114°51.607′	Desert steppe	1092	237	1.7	7.7	0.12	3

ELE: elevation, MAP: mean annual precipitation, MAT: mean annual temperature, SWC: soil water content.

**Table 2:** Hierarchical analyses of molecular variance (AMOVA) for 18 *L. chinensis* populations.

**Groups**	**Data set**	**Source of variation**	**d.f.**	**Variance components**	**Percentage variation**
None	All	Among populations within groups	17	0.274	7.28**
		Within populations	522	3.491	92.72**
Clusters	East vs. West	Among groups	1	0.239	6.16**
		Among populations within groups	16	0.149	3.85**
		Within populations	552	3.491	89.99**
Vegetation	M–T–D	Among groups	2	0.170	4.44**
		Among populations within groups	15	0.177	4.62**
		Within populations	552	3.491	90.94**
Soil pH	Nearly neutral vs. Alkaline	Among groups	1	0.013	0.33^ns^
		Among populations within groups	16	0.268	7.10**
		Within populations	552	3.491	92.57**

M: meadow, T: typical steppe and D: desert steppe, East (01–10 sites), West (11–18 sites), Nearly neutral (7.0 < pH ≤ 8.0), Alkaline (pH > 8.0). **P < 0.01, ns: not significant in each hierarchical analysis.
