# Corrigendum: What drives phenotypic divergence in *Leymus chinensis* (Poaceae) on large-scale gradient, climate or genetic differentiation?

**DOI:** 10.1038/srep46858

**Published:** 2017-06-30

**Authors:** Shan Yuan, Linna Ma, Chengyuan Guo, Renzhong Wang

Scientific Reports 6: Article number: 2628810.1038/srep26288; published online: 05 19 2016; updated: 06 30 2017

The original version of this Article contained an error in the title of the paper, where the word “drives” was incorrectly given as “drivers”. This has now been corrected in the PDF and HTML versions of the Article.

