# Supplementary material for: What drivers phenotypic divergence in Leymus chinensis (Poaceae) on large-scale gradient, climate or genetic differentiation?
Source: Sci Rep. 2016 May 19;6:26288. doi: 10.1038/srep26288 (PMC4872539; doi:10.1038/srep26288)

Supplementary Tables and Figures

What drivers phenotypic divergence in *Leymus chinensis* (Poaceae) on large-scale gradient, climate or genetic differentiation?

Shan Yuan, Linna Ma, Chengyuan Guo, Renzhong Wang*

State Key Laboratory of Vegetation and Environmental Change, Institute of Botany, the Chinese Academy of Sciences, Beijing, China

* Corresponding author

Email: wangrz@ibcas.ac.cn

Tel: +86-10-62836550 Fax: +86-10-82595962

Table S1. Population densities of 18 *L. chinensis* populations along large-scale gradient in Northeast China.

Pop: population (hereafter). D^T: total shoots density, D^R: reproductive shoots density, D^V: vegetative shoots density, RSDR, reproductive shoot differentiation ratio. Different lowercase letters indicate significant difference among populations ($P < 0.05$).

Pop.	D^T (m^{-2})	D^R (m^{-2})	D^V (m^{-2})	RSDR
01	903.4(94.4)^{a}	226.9(24.9)^{b}	676.6(112.8)^{b}	0.255(0.049)^{bc}
02	876.0(60.7)^{a}	120.6(9.6)^{c}	755.4(56.4)^{a}	0.138(0.010)^{de}
03	739.4(42.7)^{bc}	93.7(12.2)^{defg}	645.7(44.9)^{bc}	0.127(0.018)^{ef}
04	521.1(66.3)^{e}	92.0(32.2)^{defg}	429.1(69.3)^{g}	0.178(0.065)^{de}
05	636.6(36.0)^{d}	71.4(9.1)^{gh}	565.1(34.0)^{de}	0.113(0.014)^{ef}
06	768.0(80.9)^{b}	262.0(32.4)^{a}	506.0(67.4)^{ef}	0.342(0.039)^{a}
07	497.0(32.6)^{ef}	75.0(13.2)^{fgh}	422.0(37.6)^{g}	0.152(0.031)^{de}
08	556.7(94.3)^{e}	109.3(28.9)^{cde}	467.3(77.0)^{fg}	0.201(0.070)^{cd}
09	700.7(64.2)^{bcd}	88.0(11.3)^{efg}	612.7(64.9)^{bcd}	0.127(0.020)^{ef}
10	688.0(34.9)^{cd}	96.0(5.7)^{def}	592.0(33.0)^{cd}	0.140(0.008)^{de}
11	320.7(94.8)^{hi}	92.0(14.8)^{defg}	228.7(89.5)^{i}	0.321(0.153)^{a}
12	430.7(20.1)^{fg}	105.3(12.9)^{cde}	325.3(8.3)^{h}	0.244(0.018)^{bc}
13	502.7(38.4)^{ef}	81.3(11.5)^{fg}	421.3(38.4)^{g}	0.162(0.026)^{de}
14	249.7(24.2)^{i}	14.9(3.0)^{j}	234.9(24.1)^{i}	0.060(0.013)^{fg}
15	386.9(42.5)^{gh}	113.1(26.1)^{cd}	273.7(34.5)^{hi}	0.292(0.056)^{ab}
16	483.4(18.4)^{ef}	56.0(11.1)^{hi}	427.4(22.0)^{g}	0.116(0.023)^{ef}
17	304.6(38.7)^{i}	37.7(6.5)^{i}	266.9(42.7)^{hi}	0.127(0.033)^{ef}
18	283.4(49.9)^{i}	8.6(2.8)^{j}	274.9(48.8)^{hi}	0.030(0.010)^{g}
Mean	547.7(212.1)	93.2(61.6)	455.6(175.8)	0.166(0.095)

Table S2 Leaf and seed phenotypes measured from 18 populations in wild (W) and transplantation (T). FW: fresh weight, TW: turgid fresh weight, DW: dry weight, LMA: leaf mass per area. M: mean. *: $P < 0.05$, **: $P < 0.01$ between wild and transplantation populations in each site for each parameter.

		Reproductive height (cm)	Leaf length (cm)	Leaf width (cm)	Leaf area (cm^2)	FW (g)	TW (g)	DW (g)	LMA (mg cm^{-2})	Spike length (cm)	Seed weight (g)
1	W	56.75±5.21	17.91±2.09	0.48±0.07	6.8±0.9**	0.138±0.03	0.151±0.03	0.069±0.02	10.200±2.474**	11.25±0.69	1.75±0.29
	T		24.65±2.06	0.55±0.11	8.4±1.7	0.154±0.04	0.183±0.05	0.062±0.02	7.369±0.777		
2	W	49.77±3.24	17.46±2.99	0.54±0.10**	6.5±1.6**	0.130±0.04**	0.141±0.04**	0.054±0.02*	9.317±2.53**	11.25±0.61	2.11±0.41
	T		23.87±1.82	0.62±0.09	9.3±1.9	0.181±0.02	0.219±0.02	0.069±0.01	7.765±2.237		
3	W	62.24±5.63	20.55±3.03	0.52±0.10**	6.6±1.8**	0.170±0.06**	0.180±0.06**	0.071±0.03**	10.765±2.78**	12.23±0.81	2.47±0.42
	T		30.50±1.96	0.64±0.06	11.3±3.4	0.260±0.04	0.306±0.05	0.099±0.02	9.478±3.149		
4	W	65.99±4.22	21.12±3.98	0.57±0.09	7.5±1.4**	0.190±0.06	0.206±0.06	0.076±0.03	10.198±2.33**	11.64±0.64	2.21±0.33
	T		23.96±1.82	0.57±0.06	9.5±1.9	0.177±0.02	0.194±0.03	0.070±0.01	7.560±1.691		
5	W	61.08±6.38	23.57±2.50	0.49±0.06*	7.1±1.6**	0.168±0.04**	0.187±0.04**	0.063±0.02**	8.818±2.695	12.13±0.86	2.80±0.48
	T		30.51±1.75	0.55±0.05	9.3±1.8	0.236±0.02	0.278±0.02	0.094±0.01	10.420±2.290		
6	W	59.18±4.23	21.15±3.15	0.66±0.16	8.5±2.8	0.218±0.08	0.236±0.08	0.089±0.03	10.510±1.445*	11.97±0.73	2.35±0.72
	T		22.31±1.33	0.70±0.10	10.1±2.6	0.190±0.03	0.226±0.03	0.077±0.01	8.081±2.425		
7	W	66.25±4.29	22.45±2.40	0.71±0.10**	9.4±2.7*	0.259±0.07**	0.281±0.08**	0.098±0.03**	10.355±1.093	12.92±0.7	2.70±0.41
	T		26.94±3.04	0.86±0.09	12.1±4.4	0.358±0.05	0.398±0.06	0.137±0.02	12.893±5.785		
8	W	65.61±7.21	20.41±2.54	0.62±0.07**	6.3±1.5**	0.211±0.04**	0.241±0.04**	0.074±0.02	11.890±2.05**	13.30±0.78	2.76±0.64
	T		26.26±2.04	0.79±0.13	13.0±5.2	0.264±0.06	0.304±0.05	0.091±0.02	8.065±3.570		
9	W	53.99±4.29	21.49±3.14	0.60±0.10	8.8±2.4**	0.197±0.05	0.223±0.06	0.094±0.03**	10.700±5.37**	13.87±0.71	1.98±0.48
	T		25.79±3.84	0.58±0.07	9.8±2.0	0.182±0.01	0.196±0.01	0.074±0.01	7.958±2.082		
10	W	73.40±6.22	20.66±3.67	0.51±0.05**	6.1±2.1**	0.147±0.04**	0.161±0.05**	0.067±0.02**	10.950±3.099	12.23±0.57	2.35±0.57

	T		29.29±1.56	0.65±0.10	12.5±4.7	0.299±0.04	0.345±0.06	0.109±0.02	10.195±5.060		
11	W	65.16±4.32	25.31±2.84	0.66±0.07	7.1±2.1**	0.267±0.05*	0.308±0.06*	0.077±0.03*	10.878±3.13**	12.00±0.55	2.47±0.51
	T		26.85±2.05	0.67±0.08	11.5±3.6	0.230±0.03	0.254±0.03	0.085±0.01	8.188±3.160		
12	W	59.49±4.56	21.88±1.51	0.74±0.09*	5.1±2.7**	0.196±0.04**	0.218±0.05**	0.059±0.02**	11.445±3.581	12.10±30.59	2.20±0.45
	T		26.13±4.80	0.64±0.12	12.3±4.8	0.258±0.04	0.282±0.05	0.092±0.01	8.694±4.097		
13	W	65.49±5.28	20.77±2.03	0.71±0.07	8.0±1.3**	0.223±0.05	0.235±0.05*	0.100±0.02	12.423±3.04**	12.48±0.54	2.31±0.47
	T		26.49±2.05	0.72±0.07	12.6±4.7	0.258±0.05	0.290±0.06	0.092±0.01	8.579±4.288		
14	W	52.34±3.65	17.81±2.42	0.77±0.14	8.7±2.1**	0.236±0.07*	0.248±0.07**	0.107±0.03	12.255±1.808	10.70±0.62	2.12±0.34
	T		26.98±2.42	0.76±0.08	13.1±5.1	0.294±0.03	0.324±0.03	0.106±0.01	9.592±4.129		
15	W	62.11±6.92	20.25±2.81	0.67±0.10	7.6±1.6**	0.239±0.08	0.257±0.09	0.095±0.03	12.650±3.78**	10.32±0.65	2.15±0.44
	T		30.18±1.66	0.60±0.08	12.1±4.6	0.250±0.06	0.290±0.08	0.098±0.02	9.099±3.754		
16	W	58.22±3.55	19.45±1.96	0.6±0.080**	6.7±1.3**	0.191±0.05**	0.198±0.05**	0.083±0.02**	12.415±2.020*	9.86±0.38	1.80±0.35
	T		28.98±2.01	0.72±0.10	11.9±4.0	0.248±0.05	0.292±0.05	0.100±0.02	9.412±3.594		
17	W	39.38±5.21	15.94±2.23	0.66±0.07	6.8±1.6**	0.186±0.05**	0.193±0.05**	0.080±0.02**	11.705±1.278	8.04±0.52	1.53±0.45
	T		32.62±2.23	0.68±0.07	14.5±6.6	0.302±0.03	0.350±0.03	0.113±0.01	9.763±5.148		
18	W	40.81±3.29	17.38±3.28	0.61±0.07	5.2±1.2**	0.155±0.03**	0.164±0.04**	0.064±0.01**	12.320±1.364	7.18±0.42	1.43±0.38
	T		30.53±1.94	0.65±0.07	12.2±4.5	0.282±0.05	0.327±0.06	0.107±0.02	10.023±4.408		
M	W	58.58±5.71	20.86±3.54	0.62±0.12**	7.2±2.4**	0.196±0.07**	0.213±0.07**	0.078±0.03**	11.094±3.60**	11.42±0.63	2.19±0.60
	T		27.38±3.56	0.66±0.12	11.4±4.1	0.246±0.06	0.281±0.07	0.093±0.02	9.063±3.719		

Table S3 Genetic statistics of 18 *L. chinensis* populations along the longitudinal gradient. NA: alleles by Spagedi, NAe: Effective alleles, AR (k=30): Allelic richness (expected number of alleles among 30 gene copies), H_E: expected heterozygosity, I: Shannon-Wiener diversity Index. E: genotype evenness. StDev: standard deviation.

Pop.	NA	NAe	AR	H_E	I	E
01	3.7	3.1	3.6	0.619	1.110	0.098
02	3.5	3.1	3.5	0.634	1.111	0.099
03	3.5	3.2	3.5	0.659	1.151	0.104
04	3.7	3.0	3.6	0.607	1.089	0.097
05	3.5	2.9	3.4	0.624	1.089	0.095
06	3.5	2.9	3.5	0.598	1.056	0.094
07	3.5	3.1	3.4	0.640	1.116	0.099
08	3.5	2.9	3.4	0.604	1.058	0.095
09	3.5	3.1	3.5	0.638	1.121	0.100
10	3.5	3.2	3.5	0.656	1.145	0.103
11	3.3	2.5	3.1	0.541	0.916	0.080
12	2.9	2.4	2.9	0.552	0.900	0.079
13	3.1	2.8	3.0	0.608	1.012	0.089
14	3.0	2.7	3.0	0.595	0.985	0.087
15	3.3	2.6	3.1	0.567	0.966	0.083
16	3.3	2.6	3.2	0.571	0.980	0.086
17	3.3	2.8	3.2	0.606	1.035	0.090
18	3.1	2.6	3.1	0.589	0.982	0.085
Mean	3.4	2.9	3.3	0.606	1.046	0.092
StDev	0.2	0.3	0.2	0.034	0.077	0.008

Table S4 Pairwise genetic distance among 18 populations based on 15 microsatellite markers.

Pop.	01	02	03	04	05	06	07	08	09	10	11	12	13	14	15	16	17	18
01	0.000																	
02	0.120																	
03	0.086	0.048																
04	0.037	0.153	0.120															
05	0.098	0.087	0.068	0.110														
06	0.044	0.168	0.110	0.039	0.125													
07	0.096	0.088	0.065	0.118	0.089	0.125												
08	0.070	0.153	0.137	0.062	0.148	0.061	0.100											
09	0.060	0.063	0.048	0.073	0.054	0.096	0.050	0.081										
10	0.108	0.104	0.085	0.130	0.094	0.139	0.069	0.138	0.076									
11	0.189	0.382	0.325	0.175	0.242	0.201	0.235	0.193	0.225	0.238								
12	0.210	0.363	0.330	0.208	0.275	0.238	0.212	0.195	0.219	0.219	0.079							
13	0.216	0.276	0.228	0.205	0.215	0.240	0.194	0.247	0.178	0.166	0.144	0.120						
14	0.201	0.300	0.229	0.176	0.209	0.191	0.207	0.236	0.183	0.183	0.125	0.153	0.064					
15	0.178	0.356	0.289	0.177	0.299	0.156	0.243	0.185	0.249	0.239	0.094	0.136	0.140	0.133				
16	0.162	0.352	0.287	0.158	0.278	0.142	0.229	0.181	0.236	0.215	0.079	0.105	0.114	0.120	0.032			
17	0.150	0.317	0.264	0.147	0.234	0.151	0.201	0.168	0.201	0.189	0.059	0.062	0.099	0.095	0.055	0.030		
18	0.232	0.380	0.275	0.212	0.271	0.215	0.268	0.288	0.247	0.235	0.134	0.164	0.082	0.041	0.125	0.099	0.098	0.000

Table S5 Pairwise genetic differentiation (F_{ST}, lower triangular) and gene flow (Nm, upper triangular) among 18 populations based on 15 microsatellite markers.

Pop.	01	02	03	04	05	06	07	08	09	10	11	12	13	14	15	16	17	18
01	0	7.82	11.11	24.75	9.01	18.98	9.75	12.25	15.38	9.01	4.06	3.78	4.14	4.38	4.56	5.07	5.85	3.78
02	0.031	0	22.48	6.00	10.62	5.31	11.11	5.85	15.38	9.75	2.25	2.41	3.43	3.13	2.53	2.56	3.08	2.53
03	0.022	0.011	0	7.82	14.46	8.08	15.38	6.89	20.58	12.91	2.66	2.73	4.30	4.14	3.18	3.22	3.72	3.48
04	0.010	0.040	0.031	0	8.08	22.48	7.82	13.64	12.91	7.33	4.30	3.72	4.30	4.75	4.47	4.96	5.85	3.99
05	0.027	0.023	0.017	0.030	0	6.89	10.62	6.00	17.61	10.62	3.22	3.00	4.21	4.30	2.92	3.08	3.92	3.32
06	0.013	0.045	0.030	0.011	0.035	0	7.10	13.64	9.01	6.51	3.72	3.27	3.66	4.38	4.96	5.56	5.56	3.85
07	0.025	0.022	0.016	0.031	0.023	0.034	0	9.01	20.58	15.38	3.43	3.92	4.85	4.47	3.54	3.78	4.65	3.48
08	0.020	0.041	0.035	0.018	0.040	0.018	0.027	0	11.11	6.69	3.85	3.92	3.60	3.66	4.30	4.38	5.07	3.04
09	0.016	0.016	0.012	0.019	0.014	0.027	0.012	0.022	0	13.64	3.54	3.78	5.19	4.96	3.48	3.66	4.65	3.66
10	0.027	0.025	0.019	0.033	0.023	0.037	0.016	0.036	0.018	0	3.48	3.85	5.70	5.07	3.72	4.14	5.07	3.99
11	0.058	0.100	0.086	0.055	0.072	0.063	0.068	0.061	0.066	0.067	0	8.37	5.07	5.70	7.33	8.68	11.66	5.31
12	0.062	0.094	0.084	0.063	0.077	0.071	0.060	0.060	0.062	0.061	0.029	0	6.33	4.85	5.07	6.69	12.25	4.47
13	0.057	0.068	0.055	0.055	0.056	0.064	0.049	0.065	0.046	0.042	0.047	0.038	0	13.64	5.56	6.89	8.68	9.75
14	0.054	0.074	0.057	0.050	0.055	0.054	0.053	0.064	0.048	0.047	0.042	0.049	0.018	0	5.70	6.33	8.68	20.58
15	0.052	0.090	0.073	0.053	0.079	0.048	0.066	0.055	0.067	0.063	0.033	0.047	0.043	0.042	0	24.75	13.64	6.16
16	0.047	0.089	0.072	0.048	0.075	0.043	0.062	0.054	0.064	0.057	0.028	0.036	0.035	0.038	0.010	0	27.53	7.82
17	0.041	0.075	0.063	0.041	0.060	0.043	0.051	0.047	0.051	0.047	0.021	0.020	0.028	0.028	0.018	0.009	0	8.37
18	0.062	0.090	0.067	0.059	0.070	0.061	0.067	0.076	0.064	0.059	0.045	0.053	0.025	0.012	0.039	0.031	0.029	0

Table S6. Characterization of 15 microsatellite loci.

No.	*Leymus* SSR ID	Expected amplicon size	Motif	Forward primer	Reverse primer
1	Ltc0044	140	CAC	CTTCCCTGAGCCTGGACCTG	GAGTGGCATCATGAAGTTCTCCTC
2	Ltc0104	158	GCC	CACCTCTCTCGCTTCTTCCTCC	GGAGGTGAGCGGTGATTCAATAGT
3	GWM382	86	GA	GTCAGATAACGCCGTCCAAT	CTACGTGCACCACCATTTTG
4	Ltc0157	116	CAA	GCAATGAACACTGAATCAATCGAG	CGTGTGAGACTCATCGATGTTACC
5	GDM068	136	TC	GCCTGACCACTCCCATAAAA	TCGGAAGGGGGACTATACAA
6	Ltc1100	146	AGT	ACAACAGCCAAGCTAAGGTTTCAA	CGTCACCTAGCACCAATGTGTTTA
7	Ltc0182	150	TCT	ATGGTACAAGACTTACCCCACAGG	GCTCATCAAGAGCAACTGAACAGA
8	Ltc1179	133	CAA	GGTCAATGAACAAGCTGTACAAACC	CAACATCCATCTCATGCTACTTGC
9	Ltc1547	126	CA	GTTGGGCCAAGAGACTAGAAGACA	GATCCTACGGACGAGAAGATGGT
10	Ltc1570	85	TC	CACGCAAGAACACAATACAGCAT	AAAGTTTGGGACTTTCTTCTTGGC
11	Ltc0084	159	CGG	GCTGTGCGACTACCTGAACTCC	CAGAGGAACTGGAACAAGCACTC
12	Ltc0520	124	CTG	CATCGTCACGGATGAAGAACTG	AATGGCGACAGTATGTTCAGTGAT
13	Ltc0621	121	ACACA	TTCATCGATGCAACAAAAACAAAC	CCTGAGACGCTACAAAGACGTGTA
14	Ltc1369	139	GTC	ATCTGCGCTGGAGTACTGATGG	CCACCTTGAGGCCCTTGAAC
15	Ltc1514	155	AG	GAAATACAAGCCTGATGGATGGAG	CAGAGTACATCAAACGGACAGCAT

Fig. S1 Map of sampling sites. The map was generated by ArcGIS.9.3.SLX (http://www.esri.com/software/arcgis/).

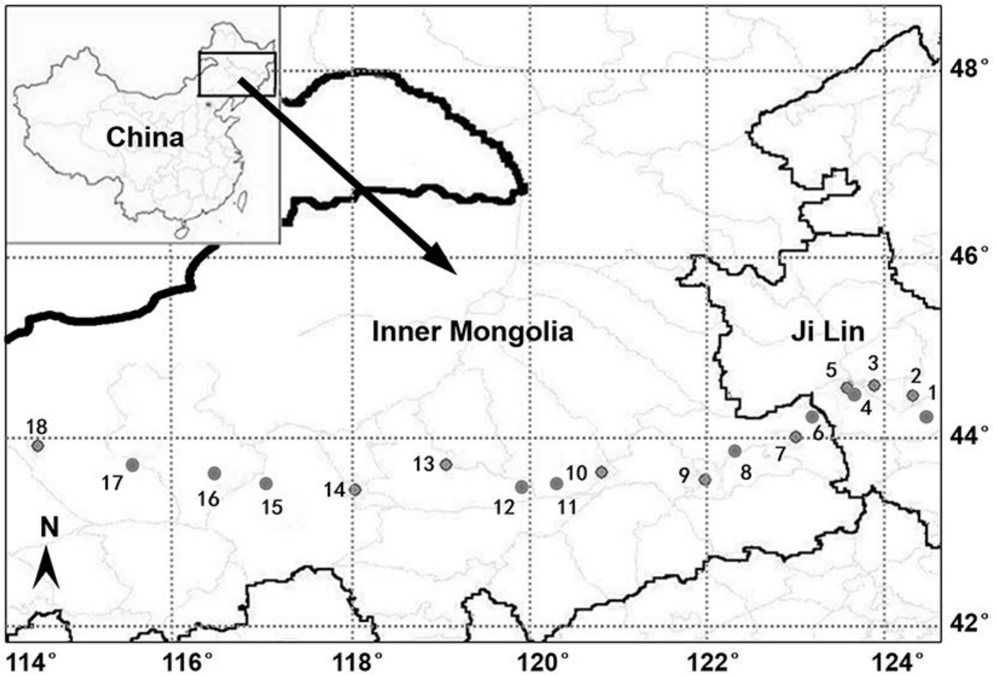

Fig. S2 Correlations of leaf length and spike length with mean annual precipitation (MAP) (a, d), elevation (ELE) (b, e), and mean annual temperature (MAT) (c, f).

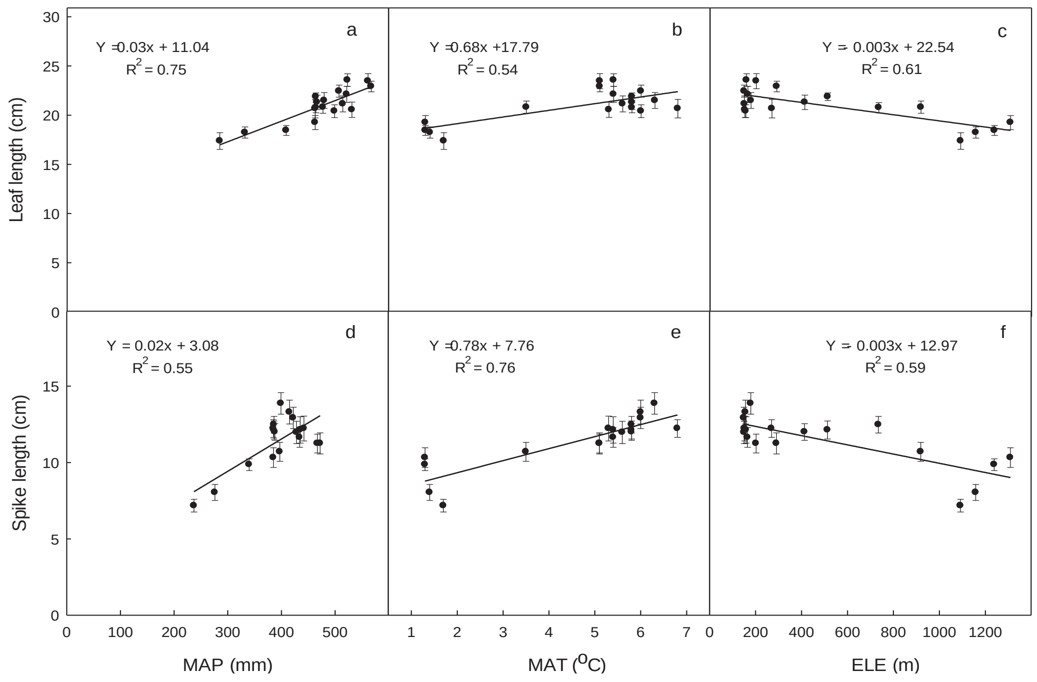

Fig. S3 The estimated mean logarithmic likelihood of K-values ranging from 1-18 with 5 replicates for each K.

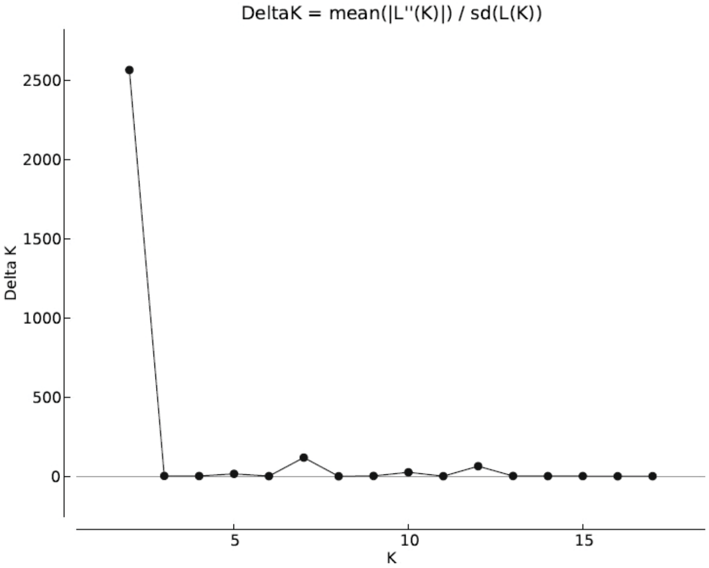

Fig. S4 Bayesian assignment results of model based clustering (K = 2-5) of 18 *L. chinensis* populations based on microsatellite data set.

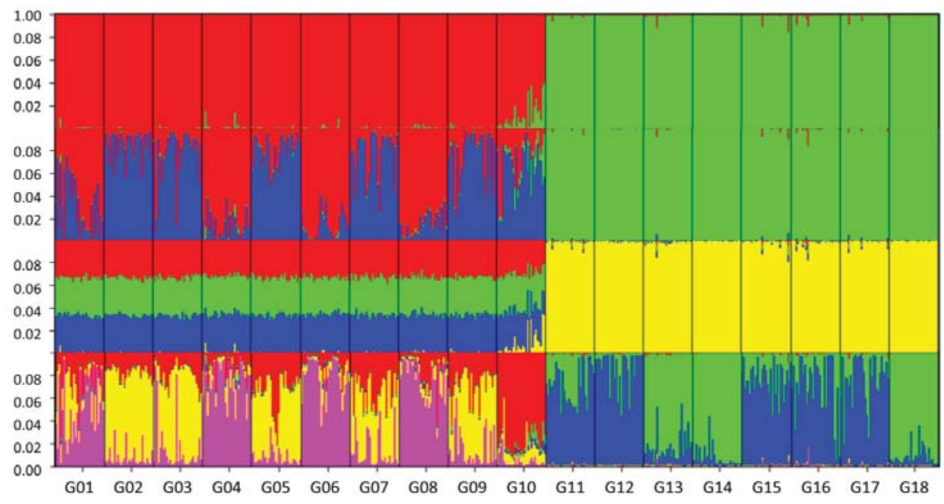

Fig. S5 Scatter plots of pairwise Nei's genetic distance (1978) and geographical distance (km) in *L. chinensis* populations.

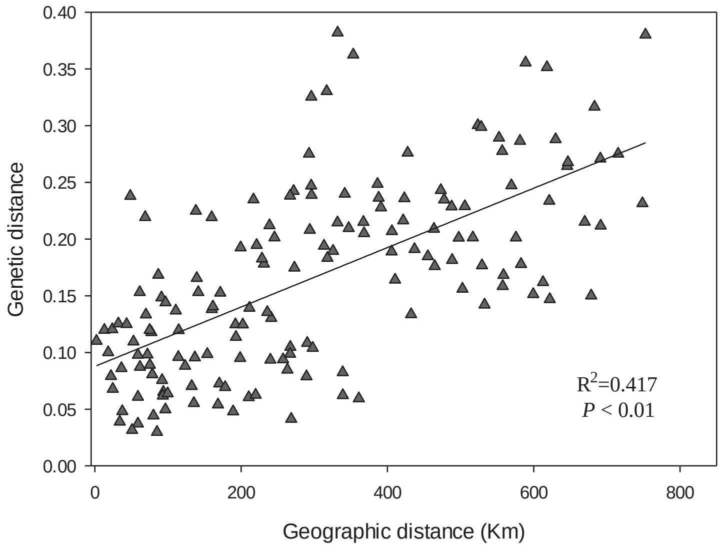

Supplement: Supplementary Information [file srep26288-s1.pdf]
